# Possible Alterations of Local Gravitational Field Inside a Superconductor

**DOI:** 10.3390/e23020193

**Published:** 2021-02-05

**Authors:** Giovanni Alberto Ummarino, Antonio Gallerati

**Affiliations:** 1 Dipartimento di Scienza Applicata e Tecnologia, Politecnico di Torino, Corso Duca Degli Abruzzi 24, 10129 Torino, Italy; giovanni.ummarino@polito.it; 2 Moscow Engineering Physics Institute, National Research Nuclear University MEPhI, Kashirskoe hwy 31, 115409 Moscow, Russia; 3 Istituto Nazionale di Fisica Nucleare, Sezione di Torino, via Pietro Giuria 1, 10125 Torino, Italy

**Keywords:** gravitation, superconductivity, Ginzburg–Landau equations, gravito-Maxwell formalism, gravity-superfluid interplay

## Abstract

We calculate the possible interaction between a superconductor and the static Earth’s gravitational fields, making use of the gravito-Maxwell formalism combined with the time-dependent Ginzburg–Landau theory. We try to estimate which are the most favorable conditions to enhance the effect, optimizing the superconductor parameters characterizing the chosen sample. We also give a qualitative comparison of the behavior of high–$T_{c}$ and classical low–$T_{c}$ superconductors with respect to the gravity/superfluid interplay.

## 1. Introduction

The study of possible gravitational effects on superconductors is more than 50 years old and started with the seminal paper of DeWitt [1]. In the following years, there has been a fair amount of scientific literature on the subject [2,3,4,5,6,7,8,9,10,11,12,13,14,15,16,17,18,19,20,21], but it was only after the 1992 Podkletnov’s reported effect [22,23] that experimental, laboratory configurations were proposed to detect the interaction.

Theoretical interpretations of the interplay between the condensate and the local gravitational field were produced in 1996 exploiting the framework of quantum gravity [24], showing how a suitable Lagrangian coupling of the superfluid can determine a gravitational interaction with the condensate and consequent localized slight instabilities [25,26]. Although being a solid and elegant formulation offering a general, theoretical explanation for the described interplay, the quantum gravity approach involves a formalism that makes it hard to extract quantitative predictions.

Parallel to DeWitt (and related) studies about gravity/supercondensate coupling, other theoretical [27,28] and experimental [29,30,31] research studies were conducted about electric-type fields induced in conductors by the presence of the gravitational field, analyzing the importance of the internal structure of special classes of solids and fluids when gravity is taken into account. Those research studies also inspired other recent papers that focus on various relevant aspects of the behavior of superconductors interacting with gravitational waves [32,33,34].

One of the results of the above studies was the introduction of a fundamental, generalized electric-like field, featuring an electrical component and a gravitational one. In the following, we are going to extend those results making use of the *gravito-Maxwell formalism* [35,36,37,38,39]. In particular, we will see that the latter approach can provide a solid framework where to obtain a generalized form for the electric/magnetic fields, involved in quantum effects originating from the interaction with the weak gravitational background. On the other side, the formalism also turns out to be powerful in the study of gravity/superconductivity interplay, since the formal analogy between the Maxwell and weak gravity equations allows us to use the Ginzburg–Landau theory for the microscopic description of the interaction. We will in fact analyze how the weak local gravitational field can be affected by the presence of the superfluid condensate, writing explicit time-dependent Ginzburg–Landau equations for the superconductor order parameter.

With respect to our previous analysis [35], we will perform new calculations in a different gauge and this will lead us to clearer and deeper conclusions on the interpretation of the conjectured effect. We will also analyze which parameters could be optimized to enhance the interaction, choosing appropriate conditions and sample characteristics.

## 2. Generalized Gravito-Maxwell Equations

Let us consider a nearly–flat spacetime configuration (weak, static gravitational field approximation) so that the metric can be expanded as:

(1) $$g_{\mu \nu }\;≃\;\eta_{\mu \nu }+h_{\mu \nu }\;,$$

where the symmetric tensor $h_{\mu \nu }$ is a small perturbation of the constant, flat Minkowski metric in the mostly plus convention, $\eta_{\mu \nu }=\mathrm{diag}\left(-1,+1,+1,+1\right)$. The inverse metric, in linear approximation, is given by

(2) $$g^{\mu \nu }\;≃\;\eta^{\mu \nu }\;-\;h^{\mu \nu }\;,$$

while the metric determinant can be expanded as

(3) $$\begin{array}{c} g\;=\;\det\left[g_{\mu \nu }\right]\;=\;\varepsilon^{\mu \nu \rho \sigma }g_{1\mu }g_{2\nu }g_{3\rho }g_{4\sigma }\;≃\;-1-h\;\;\Rightarrow \;\;\sqrt{-g}\;≃\;1+\frac{1}{2}\;h\;,\;\; \end{array}$$

where $h={h^{\sigma }}_{\;\;\sigma }$.

### 2.1. Generalizing Maxwell Equations

If we consider an inertial coordinate system, to linear order in $h_{\mu \nu }$, the connection is expanded as

(4) $${\Gamma^{\lambda }}_{\;\mu \nu }\;≃\;\frac{1}{2}\;\eta^{\lambda \rho }\;\left(\partial_{\mu }h_{\nu \rho }+\partial_{\nu }h_{\rho \mu }-\partial_{\rho }h_{\mu \nu }\right)\;.$$

The Riemann tensor is defined as:

(5) $$\begin{array}{cc} {R^{\sigma }}_{\;\mu \lambda \nu } & \;=\;\partial_{\lambda }{\Gamma^{\sigma }}_{\;\mu \nu }-\partial_{\nu }{\Gamma^{\sigma }}_{\;\mu \lambda }+{\Gamma^{\sigma }}_{\;\rho \lambda }\;{\Gamma^{\rho }}_{\;\nu \mu }-{\Gamma^{\sigma }}_{\;\rho \nu }\;{\Gamma^{\rho }}_{\;\lambda \mu }\;, \end{array}$$

while the Ricci tensor is given by the contraction

(6) $$R_{\mu \nu }\;=\;R_{\;\;\mu \sigma \nu }^{\sigma }\;,$$

and, to linear order in $h_{\mu \nu }$, it reads

(7) Rμν≃∂σΓσμν+∂μΓσσν+ΓΓ−ΓΓ=12∂μ∂ρhνρ+∂ν∂ρhμρ−12∂ρ∂ρhμν−12∂μ∂νh=∂ρ∂(μhν)ρ−12∂2hμν−12∂μ∂νh,

having used Equation (4).

The Einstein equations have the form [40]:

(8) $$R_{\mu \nu }-\frac{1}{2}\;g_{\mu \nu }\;R\;=\;8\pi G\;T_{\mu \nu }\;,$$

where $R=g^{\mu \nu }R_{\mu \nu }$ is the Ricci scalar. In first-order approximation, we can write

(9) $$\frac{1}{2}\;g_{\mu \nu }\;R\;≃\;\frac{1}{2}\;\eta_{\mu \nu }\;\eta^{\rho \sigma }R_{\rho \sigma }\;=\;\frac{1}{2}\;\eta_{\mu \nu }\;\left(\partial^{\rho }\partial^{\sigma }h_{\rho \sigma }-\partial^{2}h\right)\;,$$

having used Equation (7), and the left-hand side of (8) turns out to be

(10) $$\begin{array}{c} R_{\mu \nu }-\frac{1}{2}\;g_{\mu \nu }\;R\;≃\;\partial^{\rho }\partial_{(\mu }h_{\nu )\rho }-\frac{1}{2}\;\partial^{2}h_{\mu \nu }-\frac{1}{2}\;\partial_{\mu }\partial_{\nu }h-\frac{1}{2}\;\eta_{\mu \nu }\left(\partial^{\rho }\partial^{\sigma }h_{\rho \sigma }-\partial^{2}h\right)\;. \end{array}$$

Now, we introduce the symmetric traceless tensor

(11) $${\bar{h}}_{\mu \nu }\;=\;h_{\mu \nu }-\frac{1}{2}\;\eta_{\mu \nu }\;h\;,$$

so that the above (10) can be rewritten as

(12) $$\begin{array}{cc} R_{\mu \nu }-\frac{1}{2}g_{\mu \nu }\;R\;≃\; & \frac{1}{2}\left(\partial^{\rho }\partial_{\mu }{\bar{h}}_{\nu \rho }+\partial^{\rho }\partial_{\nu }{\bar{h}}_{\mu \rho }-\partial^{\rho }\partial_{\rho }{\bar{h}}_{\mu \nu }-\eta_{\mu \nu }\;\partial^{\rho }\partial^{\sigma }{\bar{h}}_{\rho \sigma }\right)=\;\partial^{\rho }\partial_{[\nu }{\bar{h}}_{\rho ]\mu }+\partial^{\rho }\partial^{\sigma }\eta_{\mu [\sigma }\;{\bar{h}}_{\nu ]\rho }\;\;\;\\ =\; & \partial^{\rho }\left(\partial_{[\nu }{\bar{h}}_{\rho ]\mu }+\partial^{\sigma }\eta_{\mu [\rho }\;{\bar{h}}_{\nu ]\sigma }\;\right). \end{array}$$

Then, we define the tensor

(13) $$G_{\mu \nu \rho }\;\equiv \;\partial_{[\nu }{\bar{h}}_{\rho ]\mu }+\partial^{\sigma }\eta_{\mu [\rho }\;{\bar{h}}_{\nu ]\sigma }\;,$$

so that the Einstein equations can be finally recast in the compact form:

(14) $$\partial^{\rho }G_{\mu \nu \rho }\;=\;8\pi G\;T_{\mu \nu }\;.$$

#### 2.1.1. Gauge Fixing

We now consider the *harmonic coordinate condition*, expressed by the relation [40]:

(15) $$\partial_{\mu }\left(\sqrt{-g}\;g^{\mu \nu }\right)=0\;\;\Leftrightarrow \;\;□x^{\mu }=0\;,$$

that in turn can be rewritten in the form

(16) $$g^{\mu \nu }\;{\Gamma^{\lambda }}_{\;\mu \nu }\;=\;0\;,$$

also known as *De Donder gauge*. The requirement of the above coordinate condition (15) plays then the role of a gauge fixing. Imposing the above (16) and using Equations (1) and (4), in linear approximation, we find:

(17) $$0\;≃\;\frac{1}{2}\;\eta^{\mu \nu }\eta^{\lambda \rho }\left(\partial_{\mu }h_{\nu \rho }+\partial_{\nu }h_{\rho \mu }-\partial_{\rho }h_{\mu \nu }\right)\;=\;\partial_{\mu }h^{\mu \lambda }-\frac{1}{2}\;\partial^{\lambda }h\;,$$

that is, we have the condition

(18) $$\partial_{\mu }h^{\mu \nu }≃\frac{1}{2}\;\partial^{\nu }h\;\;\Leftrightarrow \;\;\partial^{\mu }h_{\mu \nu }≃\frac{1}{2}\;\partial_{\nu }h\;.$$

Now, one also has

(19) $$\partial^{\mu }h_{\mu \nu }\;=\;\partial^{\mu }\left({\bar{h}}_{\mu \nu }+\frac{1}{2}\;\eta_{\mu \nu }h\right)\;=\;\partial^{\mu }{\bar{h}}_{\mu \nu }+\frac{1}{2}\;\partial_{\nu }h\;,$$

and, using Equation (18), we find the so-called *Lorentz gauge condition*:

(20) $$\partial^{\mu }{\bar{h}}_{\mu \nu }\;≃\;0\;.$$

The above relation further simplifies expression (13) for $G_{\mu \nu \rho }$, which takes the very simple form

(21) $$G_{\mu \nu \rho }\;≃\;\partial_{[\nu }{\bar{h}}_{\rho ]\mu }\;,$$

and verifies also the relation

(22) $$\partial_{\left[\lambda \right]}G_{\left[0|\mu \nu \right]}\;=\;0\;\;\Rightarrow \;\;G_{0\mu \nu }\propto \partial_{\mu }A_{\nu }-\partial_{\nu }A_{\mu }\;,$$

implying the existence of a potential (see next paragraph).

#### 2.1.2. Gravito-Maxwell Equations

Now, let us define the fields (for the sake of simplicity, we initially set the physical charges e=m=1)

(23) $$\begin{array}{cc} E_{g} & \;\equiv \;E_{i}\;=\;-\;\frac{1}{2}\;G_{00i}\;=\;-\;\frac{1}{2}\;\partial_{[0}{\bar{h}}_{i]0}\;, \end{array}$$

(24) $$\begin{array}{cc} A_{g} & \;\equiv \;A_{i}\;=\;\frac{1}{4}\;{\bar{h}}_{0i}\;, \end{array}$$

(25) $$\begin{array}{cc} B_{g} & \;\equiv \;B_{i}\;=\;\frac{1}{4}\;{\varepsilon_{i}}^{jk}\;G_{0jk}\;, \end{array}$$

where i=1,2,3, and

(26) $$G_{0ij}\;=\;\partial_{[i}{\bar{h}}_{j]0}\;=\;\frac{1}{2}\left(\partial_{i}{\bar{h}}_{j0}-\partial_{j}{\bar{h}}_{i0}\right)\;=\;4\;\partial_{[i}A_{j]}\;.$$

One can immediately see that

(27) $$\begin{array}{cc} B_{g} & \;=\;\frac{1}{4}\;{\varepsilon_{i}}^{jk}\;4\;\partial_{[j}A_{k]}\;=\;{\varepsilon_{i}}^{jk}\;\partial_{j}A_{k}=\nabla \times A_{g}\;,\\ \; & \;⟹\;\nabla \cdot B_{g}\;=\;0\;. \end{array}$$

Then, one also has

(28) $$\nabla \cdot E_{g}\;=\;\partial^{i}E_{i}\;=\;-\partial^{i}\frac{G_{00i}}{2}\;=\;-8\pi G\;\frac{T_{00}}{2}\;=\;4\pi G\;\rho_{g}\;,$$

using Equation (14) and having defined $\rho_{g}\equiv -T_{00}$.

If we consider the curl of $E_{g}$, we obtain

(29) $$\begin{array}{cc} \nabla \times E_{g} & \;=\;{\varepsilon_{i}}^{jk}\;\partial_{j}E_{k}\;=\;-{\varepsilon_{i}}^{jk}\;\partial_{j}\frac{G_{00k}}{2}\;=\;-\frac{1}{2}\;{\varepsilon_{i}}^{jk}\;\partial_{j}\partial_{[0}{\bar{h}}_{k]0}\;\;\\ \; & \;=\;-\frac{1}{4}\;4\;\partial_{0}\;{\varepsilon_{i}}^{jk}\;\partial_{j}A_{k}\;=\;-\partial_{0}B_{i}\;=\;-\frac{\partial B_{g}}{\partial t}\;. \end{array}$$

Finally, one finds for the curl of $B_{g}$

(30) $$\begin{array}{cc} \nabla \times B_{g} & \;=\;{\varepsilon_{i}}^{jk}\;\partial_{j}B_{k}\;=\;\frac{1}{4}\;{\varepsilon_{i}}^{jk}\;{\varepsilon_{k}}^{\ell m}\;\partial_{j}G_{0\ell m}\;=\;\frac{1}{4}\left({\delta_{i}}^{\ell }\delta^{jm}-{\delta_{i}}^{m}\delta^{j\ell }\right)\partial_{j}G_{0\ell m}\;\;\\ \; & \;=\;\frac{1}{2}\;\partial^{j}G_{0ij}\;=\;\frac{1}{2}\left(\partial^{\mu }G_{0i\mu }+\partial_{0}G_{0i0}\right)\;=\;\frac{1}{2}\left(\partial^{\mu }G_{0i\mu }-\partial_{0}G_{00i}\right)\;\;\\ \; & \;=\;\frac{1}{2}\left(8\pi G\;T_{0i}-\partial_{0}G_{00i}\right)\;=\;4\pi G\;j_{i}+\frac{\partial E_{i}}{\partial t}\;=\;4\pi G\;j_{g}\;+\;\frac{\partial E_{g}}{\partial t}\;, \end{array}$$

using again Equation (14) and having defined $j_{g}\equiv j_{i}\equiv T_{0i}$.

Summarizing, once defined the fields of Equations (23) to (25) and having restored physical units, one gets the field equations:

(31) $$\begin{array}{cc} \; & \nabla \cdot E_{g}\;=\;4\pi G\;\rho_{g}\;,\\ \; & \nabla \cdot B_{g}\;=\;0\;,\\ \; & \nabla \times E_{g}\;=-\frac{\partial B_{g}}{\partial t}\;,\\ \; & \nabla \times B_{g}\;=\;\frac{4\pi G}{c^{2}}\;j_{g}\;+\;\frac{1}{c^{2}}\;\frac{\partial E_{g}}{\partial t}\;, \end{array}$$

formally equivalent to Maxwell equations, where $E_{g}$ and $B_{g}$ are the gravitoelectric and gravitomagnetic field, respectively. For instance, on the Earth’s surface, $E_{g}$ corresponds to the Newtonian gravitational acceleration, while $B_{g}$ is related to angular momentum interactions [15,41,42]. The mass current density vector $j_{g}$ can also be expressed as:

(32) $$j_{g}\;=\;\rho_{g}\;v\;,$$

where v is the velocity, and $\rho_{g}$ is the mass density.

#### 2.1.3. Gravito-Lorentz Force

Let us consider the geodesic equation for a particle in the presence of a weak gravitational field:

(33) $$\frac{d^{2}x^{\lambda }}{ds^{2}}\;+\;{\Gamma^{\lambda }}_{\;\mu \nu }\;\frac{dx^{\mu }}{ds}\;\frac{dx^{\nu }}{ds}\;=\;0\;.$$

If we consider a non-relativistic motion, the velocity of the particle can be expressed as $\frac{v_{i}}{c}≃\frac{dx^{i}}{dt}$. If we also neglect terms in the form $\frac{v_{i}\;v^{j}}{c^{2}}$ and limit ourselves to static metric configurations, we find that a geodesic equation for the particle in non-relativistic motion is written as [43,44]:

(34) $$\frac{dv}{dt}\;=\;E_{g}+v\times B_{g}\;,$$

which shows that a free falling particle is governed by the analogous of a Lorentz force produced by the gravito-Maxwell fields.

#### 2.1.4. Generalized Maxwell Equations

It is now straightforward to define generalized electric/magnetic fields, scalar and vector potentials, containing both electromagnetic and gravitational contributions, as:

(35) $$E=E_{e}+\frac{m}{e}\;E_{g}\;;\;\;B=B_{e}+\frac{m}{e}\;B_{g}\;;\;\;\phi =\phi_{e}+\frac{m}{e}\;\phi_{g}\;;\;\;A=A_{e}+\frac{m}{e}\;A_{g}\;,$$

where *m* and *e* are the electron mass and charge, respectively.

The generalized Maxwell equations then become:

(36) $$\begin{array}{cc} \; & \nabla \cdot E\;=\;\left(\frac{1}{\varepsilon_{g}}+\frac{1}{\varepsilon_{0}}\right)\;\rho \;,\\ \; & \nabla \cdot B\;=\;0\;,\\ \; & \nabla \times E\;=-\frac{\partial B}{\partial t}\;,\\ \; & \nabla \times B\;=\;\left(\mu_{g}+\mu_{0}\right)\;j\;+\;\frac{1}{c^{2}}\;\frac{\partial E}{\partial t}\;, \end{array}$$

where $\varepsilon_{0}$ and $\mu_{0}$ are the electric permittivity and magnetic permeability in the vacuum, and where we have set

(37) $$\rho_{g}\;=\;\frac{m}{e}\;\rho \;,\;\;j_{g}\;=\;\frac{m}{e}\;j\;,$$

ρ and j being the electric charge density and electric current density, respectively. The introduced vacuum gravitational permittivity $\varepsilon_{g}$ and vacuum gravitational permeability $\mu_{g}$ are defined as

(38) $$\varepsilon_{g}=\frac{1}{4\pi G}\;\frac{e^{2}}{m^{2}}\;,\;\;\mu_{g}=\frac{4\pi G}{c^{2}}\;\frac{m^{2}}{e^{2}}\;.$$

In this Section, we have then shown how to define a new set of generalized Maxwell equations for generalized electric E and magnetic B fields, in the limit of weak gravitational field. In the following, we are going to use these results to analyze the interaction between a superconducting sample and the weak, static Earth’s gravitational field.

## 3. The Model

Now, we are going to study in detail the conjectured gravity/superconductivity interplay making use of the Ginzburg–Landau formulation combined with the described gravito-Maxwell formalism. In particular, we write the Ginzburg–Landau equations for a superconducting sample in the weak, static Earth’s gravitational field. The latter is formally treated as the gravitational component of a generalized electric field, exploiting the formal analogy discussed in the previous Section 2.

### 3.1. Time-Dependent Ginzburg–Landau Formulation

Since the gravitoelectric field is formally analogous to a generalized electric field, we can use the time-dependent Ginzburg–Landau equations (TDGL) written in the form [45,46,47,48,49,50,51]:

(39) $$\begin{array}{cc} \; & \frac{\hbar^{2}}{2\;m\;D}\left(\frac{\partial }{\partial t}+\frac{2\;i\;e}{\hbar }\;\phi \right)\;\psi \;-\;a\;\psi \;+\;b\;{\left|\psi \right|}^{2}\psi \;+\;\frac{1}{2\;m}{\left(i\hbar \;\nabla +\frac{2\;e}{c}\;A\right)}^{2}\psi \;=\;0\;, \end{array}$$

(40) $$\begin{array}{cc} \; & \nabla \times \nabla \times A-\nabla \times H\;=-\frac{4\pi }{c}\;\left(j_{n}+j_{s}\right)\;, \end{array}$$

where $j_{n}$ and $j_{s}$ are expressed as

(41) $$\begin{array}{cc} j_{n} & \;=\;\sigma \left(\frac{1}{c}\;\frac{\partial A}{\partial t}+\nabla \phi \right)\;,\\ j_{s} & \;=\;\frac{e}{m}\left(i\hbar \left(\psi^{*}\;\nabla \psi -\psi \;\nabla \psi^{*}\right)+\frac{4\;e}{c}\;{\left|\psi \right|}^{2}A\right)\;, \end{array}$$

and denote the contributions related to the normal current and supercurrent densities, respectively. The TDGL Equations (39) and (40) for the variables ψ, A are derived minimizing the total Gibbs free energy of the system [52,53,54]. In the above expressions, D is the diffusion coefficient, σ is the conductivity in the normal phase, H is the applied field and the vector field A is minimally coupled to ψ. The coefficients *a* and *b* in (39) have the following form:

(42) $$a\;=\;a\left(T\right)\;=\;a_{0}\;\left(T-T_{c}\right)\;,\;\;b\;=\;b\left(T_{c}\right)\;,\;$$

$a_{0}$, *b* being positive constants, and $T_{c}$ the critical temperature of the superconductor. The boundary and initial conditions are

(43) $$\begin{array}{c} \left.\begin{array}{cc} \left(i\hbar \;\nabla \psi +\frac{2\;e}{c}\;A\;\psi \right)\cdot n=0 & \\ \nabla \times A\cdot n=H\cdot n & \\ A\cdot n=0 \end{array}\;\;\right\}\;\mathrm{on}\;\partial \Omega \times \left(0,t\right)\;,\;\;\left.\begin{array}{cc} \psi (x,0) & \;=\;\psi_{0}\left(x\right)\\ A(x,0) & \;=\;A_{0}\left(x\right) \end{array}\;\;\;\;\right\}\;\mathrm{on}\;\Omega \;,\; \end{array}$$

where ∂Ω is the boundary of a smooth and simply connected domain in $R^{N}$.

#### 3.1.1. Dimensionless TDGL

In order to write Equations (39) and (40) in a dimensionless form, the following expressions can be introduced:

(44) $$\begin{array}{cc} \; & \Psi^{2}\left(T\right)=\frac{\left|a(T)\right|}{b}\;,\;\;\xi \left(T\right)=\frac{h}{\sqrt{2\;m\;|a(T)|}}\;,\;\;\lambda \left(T\right)=\sqrt{\frac{b\;m\;c^{2}}{4\pi \;|a\left(T\right)|\;e^{2}}}\;,\;\;\kappa =\frac{\lambda (T)}{\xi (T)}\;,\;\\ \; & \tau \left(T\right)=\frac{\lambda^{2}\left(T\right)}{D}\;,\;\;\eta =\frac{4\pi \;\sigma \;D}{\varepsilon_{0}\;c^{2}}\;,\;\;\;H_{c}\left(T\right)=\sqrt{\frac{4\pi \;\mu_{0}\;{\left|a(T)\right|}^{2}}{b}}=\frac{h}{4\;e\;\sqrt{2\pi }\;\lambda \left(T\right)\;\xi \left(T\right)}\;, \end{array}$$

where λ(T), ξ(T) and $H_{c}\left(T\right)$ are the penetration depth, coherence length and thermodynamic critical field, respectively. We also define the dimensionless quantities

(45) $$x^{\prime }=\;\frac{x}{\lambda }\;,\;\;\;t^{\prime }=\;\frac{t}{\tau }\;,\;\;\;\psi^{\prime }=\;\frac{\psi }{\Psi }\;,$$

and the dimensionless fields are then written as:

(46) $$A^{\prime }\;=\;\frac{A\;\kappa }{\sqrt{2}\;H_{c}\;\lambda }\;,\;\;\;\phi^{\prime }\;=\;\frac{\phi \;\kappa }{\sqrt{2}\;H_{c}\;D}\;,\;\;\;H^{\prime }\;=\;\frac{H\;\kappa }{\sqrt{2}\;H_{c}}\;.$$

Inserting Equations (45) and (46) in Equations (39) and (40) and dropping the primes gives the dimensionless TDGL equations in a bounded, smooth and simply connected domain in $R^{N}$ [45,46]:

(47) $$\begin{array}{cc} \; & \frac{\partial \psi }{\partial t}\;+\;i\;\phi \;\psi \;+\;\kappa^{2}\left({\left|\psi \right|}^{2}-1\right)\;\psi \;+\;{\left(i\;\nabla +A\right)}^{2}\psi \;=\;0\;, \end{array}$$

(48) $$\begin{array}{cc} \; & \nabla \times \nabla \times A-\nabla \times H\;=\;-\eta \;\left(\frac{\partial A}{\partial t}+\nabla \phi \right)-\frac{i}{2\;\kappa }\left(\psi^{*}\nabla \psi -\psi \nabla \psi^{*}\right)-{\left|\psi \right|}^{2}A\;, \end{array}$$

and the boundary and initial conditions (43) become, in the dimensionless form,

(49) $$\begin{array}{c} \left.\begin{array}{cc} \left(i\;\nabla \psi +A\;\psi \right)\cdot n=0 & \\ \nabla \times A\cdot n=H\cdot n & \\ A\cdot n=0 \end{array}\;\;\;\right\}\;\mathrm{on}\;\partial \Omega \times \left(0,t\right)\;;\;\;\left.\begin{array}{cc} \psi (x,0) & \;=\;\psi_{0}\left(x\right)\\ A(x,0) & \;=\;A_{0}\left(x\right) \end{array}\;\;\;\;\right\}\;\mathrm{on}\;\Omega \;.\; \end{array}$$

### 3.2. Solving Dimensionless TDGL

Now, we will study the possible local alterations of the Earth’s gravitational field (weak uniform field) inside a superconductor. Let us consider the dimensionless form of the time-dependent Ginzburg–Landau equations in the gauge of vanishing scalar potential ϕ=0 [55]:

(50) $$\begin{array}{cc} \frac{\partial \psi }{\partial t}\; & =\;-{\left(\frac{i}{\kappa }\;\nabla +A\right)}^{\;\;2}\psi \;+\;\left(1-{\left|\psi \right|}^{2}\right)\psi \;, \end{array}$$

(51) $$\begin{array}{cc} \eta \;\frac{\partial A}{\partial t}\; & =\;-\;\nabla \times \nabla \times A\;+\;\nabla \times H-{\left|\psi \right|}^{2}\left(A-\frac{1}{\kappa }\;\nabla \theta \right)\;, \end{array}$$

where ψ≡ψ(x,t) is a complex function that we express as

(52) ψ=|ψ|exp(iθ)=Reψ+iImψ=ψ1+iψ2,

so that (50) gives two distinct equations for the real and imaginary parts $\psi_{1}$ and $\psi_{2}$.

We remark that here we have decided to use the most convenient option for subsequent calculations, since any gauge choice shall not influence any physical results, being the equations gauge-invariant. From a physical point of view, the ϕ=0 gauge is also motivated by the fact that there are no localized charges in the superconductor, while any contribution to the total gravitational field coming from the superconductor mass is irrelevant and can be neglected.

#### 3.2.1. 1-D Case

Let us now restrict to the 1-dimensional case ( ∇→∂/∂x, $A\to A_{x}\equiv A$ ). In this situation, the above TDGL Equations (50) and (51) give rise to the following equations:

(53) $$\begin{array}{cc} \frac{\partial \psi_{1}}{\partial t} & \;=\;\frac{1}{\kappa^{2}}\;\frac{\partial^{2}\psi_{1}}{{\partial x}^{2}}+\frac{2A}{\kappa }\;\frac{\partial \psi_{2}}{\partial x}+\frac{\psi_{2}}{\kappa }\;\frac{\partial A}{\partial x}\;-\psi_{1}A^{2}+\psi_{1}-\psi_{1}\left(\psi_{1}^{2}+\psi_{2}^{2}\right)\;,\\ \frac{\partial \psi_{2}}{\partial t} & \;=\;\frac{1}{\kappa^{2}}\;\frac{\partial^{2}\psi_{2}}{{\partial x}^{2}}-\frac{2A}{\kappa }\;\frac{\partial \psi_{1}}{\partial x}-\frac{\psi_{1}}{\kappa }\;\frac{\partial A}{\partial x}-\psi_{2}\;A^{2}+\psi_{2}-\psi_{2}\left(\psi_{1}^{2}+\psi_{2}^{2}\right)\;,\\ \eta \;\frac{\partial A}{\partial t} & \;=\;-\frac{1}{\kappa }\left(\psi_{2}\;\frac{\partial \psi_{1}}{\partial x}-\psi_{1}\;\frac{\partial \psi_{2}}{\partial x}\right)-\left(\psi_{1}^{2}+\psi_{2}^{2}\right)A-4\pi j_{n}\;, \end{array}$$

where $j_{n}$ indicates the normal current density.

Now, we consider a half-infinite superconductive region, where the $\vec{x}$ direction is perpendicular to superconductor surface (coinciding with the yz plane), i.e., we imagine that, for x>0, we have an empty space, while the region occupied by the material is located at x≤0. The system is immersed in a static, uniform gravitational field $E_{g}^{EXT}=-g\;{\vec{\mu }}_{x}$, where *g* is the standard gravity acceleration. We are in the gauge where, in the *dimensional* form, we can write for the gravitoelectric field inside the superconductor

(54) $$E_{g}=-\frac{\partial A_{g}\left(t\right)}{\partial t}\;,$$

while the external gravitational vector potential outside the superconductor is given by

(55) $$A_{g}^{\mathrm{EXT}}\left(t\right)=g\left(C+t\right)\;{\vec{\mu }}_{x}\;,$$

where *C* is a constant. In the 1-D *dimensionless* form, dropping the primes, we have

(56) $$A^{EXT}\;=\;\frac{m}{e}\;A_{g}^{EXT}\;\frac{\kappa }{\sqrt{2}\;H_{c}\;\lambda }\;=\;g_{⋆}\left(c_{1}+t\right)\;,$$

with

(57) $$c_{1}\;=\;\frac{C}{\tau }\;,\;\;\;\;g_{⋆}\;=\;\frac{m\;\kappa \;\lambda (T)\;g}{\sqrt{2}\;e\;D\;H_{c}\left(T\right)}\;\ll \;1\;.$$

having used relations (44).

Next, we express the $\psi_{1}$, $\psi_{2}$ and *A* fields as:

(58) $$\begin{array}{cc} \psi_{1}\left(x,t\right) & \;=\;\psi_{10}\left(x\right)+g_{⋆}\;\gamma_{1}\left(x,t\right)\;, \end{array}$$

(59) $$\begin{array}{cc} \psi_{2}\left(x,t\right) & \;=\;\psi_{20}\left(x\right)+g_{⋆}\;\gamma_{2}\left(x,t\right)\;, \end{array}$$

(60) $$\begin{array}{cc} A(x,t) & \;=\;g_{⋆}\;\beta \left(x,t\right)\;, \end{array}$$

where $\psi_{10}$ and $\psi_{20}$ represent the unperturbed system and satisfy

(61) $$\begin{array}{cc} 0 & \;=\;\frac{1}{\kappa^{2}}\;\frac{\partial^{2}\psi_{10}}{{\partial x}^{2}}+\psi_{10}-\psi_{10}\left(\psi_{10}^{2}+\psi_{20}^{2}\right)\;, \end{array}$$

(62) $$\begin{array}{cc} 0 & \;=\;\frac{1}{\kappa^{2}}\;\frac{\partial^{2}\psi_{20}}{{\partial x}^{2}}+\psi_{20}-\psi_{20}\left(\psi_{10}^{2}+\psi_{20}^{2}\right)\;. \end{array}$$

The $\psi_{10}$ and $\psi_{20}$ components satisfy the same kind of equation, and we choose to set $\psi_{20}=0$ ($\psi_{0}=\psi_{10}+i\;\psi_{20}=\psi_{10}\;\in R$), so that $\psi_{10}=\tanh\frac{\kappa x}{\sqrt{2}}$ gives the standard solution for (61) [53]. We are then left with the following set of equations:

(63) $$\begin{array}{cc} \frac{\partial \gamma_{1}}{\partial t} & \;=\;\frac{1}{\kappa^{2}}\;\frac{\partial^{2}\gamma_{1}}{{\partial x}^{2}}+\left(1-3\;\psi_{10}^{2}\right)\gamma_{1}\;, \end{array}$$

(64) $$\begin{array}{cc} \frac{\partial \gamma_{2}}{\partial t} & \;=\;\frac{1}{\kappa^{2}}\;\frac{\partial^{2}\gamma_{2}}{{\partial x}^{2}}+\left(1-\;\psi_{10}^{2}\right)\gamma_{1}-\frac{2\beta }{\kappa }\;\frac{\partial \psi_{10}}{\partial x}-\frac{\psi_{10}}{\kappa }\;\frac{\partial \beta }{\partial x}\;, \end{array}$$

(65) $$\begin{array}{cc} \eta \;\frac{\partial \beta }{\partial t} & \;=\;-\frac{1}{\kappa }\left(\gamma_{2}\;\frac{\partial \psi_{10}}{\partial x}-\psi_{10}\;\frac{\partial \gamma_{2}}{\partial x}\right)-\psi_{10}^{2}\;\beta \;, \end{array}$$

where the last (65) implies that β(x,t) does not depend on $\gamma_{1}\left(x,t\right)$. If we decide to put ourselves away from borders, we can set $\psi_{10}≃1$ in Equations (63) to (65), obtaining

(66) $$\begin{array}{cc} \frac{\partial \gamma_{1}}{\partial t} & \;≃\;\frac{1}{\kappa^{2}}\;\frac{\partial^{2}\gamma_{1}}{{\partial x}^{2}}-2\;\gamma_{1}\;, \end{array}$$

(67) $$\begin{array}{cc} \frac{\partial \gamma_{2}}{\partial t} & \;≃\;\frac{1}{\kappa^{2}}\;\frac{\partial^{2}\gamma_{2}}{{\partial x}^{2}}-\frac{1}{\kappa }\;\frac{\partial \beta }{\partial x}\;, \end{array}$$

(68) $$\begin{array}{cc} \eta \;\frac{\partial \beta }{\partial t} & \;≃\;\frac{1}{\kappa }\;\frac{\partial \gamma_{2}}{\partial x}-\beta \;, \end{array}$$

that gives for β the explicit solution

(69) $$\beta \left(x,t\right)\;=\;e^{-\frac{t}{\eta }}\;\left(b_{1}\left(x\right)+\frac{1}{\kappa \;\eta }\;\int_{0}^{t}\;\;dt\;e^{\frac{t}{\eta }}\;\frac{\partial \gamma_{2}\left(x,t\right)}{\partial x}\right)\;,$$

where $b_{1}\left(x\right)=c_{1}$, as it is implied by Equation (60) for t≃0.

Let us keep in mind that we are considering a semi-infinite superconductor whose surface is parallel to the ground and normal to the $\vec{x}$ axis (one-dimensional case) where the external vector potential is expressed as:

(70) $$A^{EXT}\left(t\right)\;=\;\left(c_{1}+t\right)\;g_{⋆}\;.$$

At the time t=0, the sample goes in the superconductive state, while we make the natural assumption that in the normal state (t<0) the material has just the standard (Newtonian) interaction with the Earth’s gravity, implying that the local gravitational field assumes the same values inside and outside the sample for t<0. We then write the following boundary conditions:

(71) $$\begin{array}{c} \begin{array}{cccccc} \; & \psi (0,t)=0\;,\;\;\; & \; & \psi \left(x,0\right)=\psi_{10}\left(x\right)\;,\;\;\; & \; & \frac{\partial \psi_{1}}{\partial x}\left(x,0\right)=0\;,\\ \; & \gamma_{1}\left(0,t\right)=0\;,\;\;\; & \; & \gamma_{1}\left(x,0\right)=0\;,\;\;\; & \; & \frac{\partial \gamma_{1}}{\partial x}\left(x,0\right)=0\;,\\ \; & \gamma_{2}\left(0,t\right)=0\;,\;\;\; & \; & \gamma_{2}\left(x,0\right)=0\;,\;\;\; & \; & \frac{\partial \gamma_{2}}{\partial x}\left(x,0\right)=0\;, \end{array} \end{array}$$

together with the condition

(72) $$\lim_{\;t\to 0}\;g_{⋆}\;\frac{\partial f}{\partial t}\beta \left(x,t\right)\;=\;g_{⋆}\;,$$

implying that the effect takes place when the superconducting phase appears.

Let us now fix the constant $c_{1}$. Using (65), we can express the relation between $E_{g}$ and β as

(73) $$\frac{E_{g}}{g_{⋆}}\;=\;-\frac{\partial \beta }{\partial t}\;=\;\frac{1}{\kappa \;\eta }\left(\gamma_{2}\;\frac{\partial \psi_{10}}{\partial x}-\psi_{10}\;\frac{\partial \gamma_{2}}{\partial x}\right)+\frac{\psi_{10}^{2}}{\eta }\;\beta \;.$$

Given the natural hypothesis that the affection of the gravitational field only exists when the material is in the superconductive state (t>0), we expect that, at initial time,

(74) $$\lim_{\;t\to 0^{+}}\;\frac{E_{g}}{g_{⋆}}\;=\;1\;,$$

while, from conditions (71), we also have

(75) $$\lim_{\;t\to 0^{+}}\;\gamma_{2}\left(x,t\right)\;=\;0\;,\;\;\lim_{\;t\to 0^{+}}\;\frac{\partial \gamma_{2}}{\partial x}\left(x,t\right)\;=\;0\;,$$

from which we get, in turn,

(76) $$1\;=\;\frac{\psi_{10}^{2}}{\eta }\;\beta \left(x,0^{+}\right)\;=\;\frac{\psi_{10}^{2}}{\eta }\;\frac{A^{\mathrm{EXT}}\left(0^{+}\right)}{g_{⋆}}\;=\;\frac{\psi_{10}^{2}}{\eta }\;c_{1}\;\;⟹\;\;c_{1}=\frac{\eta }{\psi_{10}^{2}}\;.$$

This constant is ineffective in the empty space, while it determines physical effects in the superconductive state. The above formulation shows how the described interplay should work: the external gravitational field is affected by the presence of the sample only when it goes in the superconductive state (when the vector potential starts to “feel” the presence of the superfluid). From the other side, the external gravitational vector potential seems involved in the material superconductive transition, since the external constant $c_{1}$ tends to assume a fixed value related to the properties of the superfluid entering the superconducting state.

Now, we can rewrite the explicit solution for β(x,t) away from borders $\left(\psi_{10}≃1\right)$:

(77) $$\beta \left(x,t\right)\;=\;e^{-\frac{t}{\eta }}\;\left(\eta +\frac{1}{\kappa \;\eta }\;\int_{0}^{t}\;\;dt\;e^{\frac{t}{\eta }}\;\frac{\partial \gamma_{2}\left(x,t\right)}{\partial x}\right)\;,$$

from which we get the ratio

(78) $$\frac{E_{g}}{g_{⋆}}\;=\;-\frac{\partial \beta (x,t)}{\partial t}\;=\;\frac{1}{\eta }\;e^{-\frac{t}{\eta }}\;\left(\eta +\frac{1}{\kappa \;\eta }\;\int_{0}^{t}\;\;dt\;e^{\frac{t}{\eta }}\;\frac{\partial \gamma_{2}\left(x,t\right)}{\partial x}\right)-\frac{1}{\kappa \;\eta }\frac{\partial \gamma_{2}\left(x,t\right)}{\partial x}\;.$$

## 4. Discussion

Given the explicit expression (78) for the ratio $E_{g}/g_{⋆}$, we can estimate, for $t≃0^{+}$, the value of gravitational field inside the superconductor:

(79) $$t≃0^{+}\;:\;\frac{E_{g}}{g_{⋆}}=1-\frac{t}{\eta }-\frac{1}{\kappa \;\eta }\frac{\partial \gamma_{2}\left(x,0^{+}\right)}{\partial x}\;.$$

In the superconductive state, the gravitational field is modified in a way that depends on physical characteristic of the particular material. We can see from the above (79) that the involved quantities are η, κ and the spatial derivative of $\gamma_{2}$.

Let us discuss which should be the most favorable choices for the parameters to enhance the desired interaction. First of all, we would like to maximize $\frac{\partial \gamma_{2}}{\partial x}$: to do this, it is sufficient to introduce disorder in the material, induced, for instance, by means of proton irradiation or chemical doping. Then, we also want a small η parameter: being the latter proportional to the product of the diffusion coefficient times the conductivity just above $T_{c}$, it is necessary to have materials that in the normal state are bad conductors and have low Fermi energies, such as cuprates. The last parameter to optimize is a reduced value for κ, which is usually small in low–$T_{c}$ superconductors and high in cuprates. Clearly, we can see that optimizing at the same time last two parameters gives rise to contrasting effects; however, analyzing the involved values, the better choice is to maximize η, thus using a superconducting cuprate with high disorder.

It is also very important to maximize the time scale ($\tau =\lambda^{2}/D$) in order to better observe the effect. This is achieved by increasing the penetration length and reducing the diffusivity coefficient, just as it occurs in superconducting cuprates with disorder.

In Table 1 and Table 2 it is possible to see typical parameters of low (Pb) and high (YBCO) $T_{c}$ superconductors, some of which calculated at a temperature $T^{*}$ such that the quantity $\frac{T^{*}-T_{c}}{T_{c}}$ is the same in the two materials. If we go closer to $T_{c}$, it is possible to increase the effect: for example, at T=87K in the case of YBCO τ is of the order of $10^{-9}\;s$ and the reduction of the gravitational field is of the order of $10^{-7}$, having neglected the last term in Equation (79) (In high–$T_{c}$ superconductors not irradiated, we usually have low disorder, so that the spatial derivative of $\gamma_{2}$ is small; moreover, there is an additional reduction of order $10^{2}$ coming from the κ parameter at denominator.).

## 5. Concluding Remarks

We have shown how the gravito-Maxwell formalism can be instrumental in describing a gravity/superfluid interplay, when combined with the condensed matter formalism of the time-dependent Ginzburg–Landau equations. Our analysis suggests that a non-negligible interaction could be present, despite the experimental detection difficulties that may arise, especially in relation to the short time intervals in which the effect occurs. In particular, the dimensionless TDGL can provide qualitative and quantitative suggestion about the magnitude of the interaction, once chosen appropriate boundary conditions.

Clearly, proper arrangement of the experimental setup is crucial to maximize the effect. In particular, the focus should be on suitable sample geometry, material parameters and laboratory settings, so as to enhance the interaction in workable time scales [37,38,56]. It is also possible that a significant improvement comes from the presence of external electric and magnetic fields, since the latter determine the presence of moving vortices, giving rise to a possible additional affection of the local gravitational field.

## Figures and Tables

**Table 1:** YBCO vs. Pb.

	YBCO	Pb
$T_{c}$	89K	7.2 K
$T_{⋆}$	77K	6.3 K
$\xi (T_{⋆})$	$3.6\cdot 10^{-9}\;m$	1.7 · 10^−7^ m
$\lambda (T_{⋆})$	$3.3\cdot 10^{-7}\;m$	7.8 · 10^−8^ m
$\sigma^{-1}$	$4.0\cdot 10^{-7}\;\Omega \;m\;{}^{\left(*\right)}$	2.5 · 10^−9^ m Ω m (**)
$H_{c}\left(T_{⋆}\right)$	0.2Tesla	0.018 Tesla
κ	94.4	0.48
$\tau (T_{⋆})$	$3.4\cdot 10^{-10}\;s$	6.1 · 10^−15^ s
η	$1.3\cdot 10^{-2}$	6.6 · 10^3^
$g_{⋆}$	$2.0\cdot 10^{-11}$	8.2 · 10^−17^
D	$3.2\cdot 10^{-4}\;m^{2}/s$	1 m^2^/s
*ℓ*	$6.0\cdot 10^{-9}\;m$	1.7 · 10^−6^ m
$v_{F}$	$1.6\cdot 10^{5}\;m/s$	1.8 · 10^6^ m/s
	${}^{\left(*\right)}\;T\;=\;90\;K$	${}^{\left(**\right)}\;T\;=\;15\;K$

**Table 2:** (**i**): YBCO. (**ii**): Pb.

(i)			
YBCO	λ	τ	*g_⋆_*
T=0K0	$1.7\cdot 10^{-7}\;m$	$9.03\cdot 10^{-11}\;s$	2.6 · 10^−12^
T=70K	$2.6\cdot 10^{-7}\;m$	$2.1\cdot 10^{-10}\;s$	9.8 · 10^−12^
T=77K	$3.3\cdot 10^{-7}\;m$	$3.4\cdot 10^{-10}\;s$	2 · 10^−11^
T=87K	$8\cdot 10^{-7}\;m$	$2\cdot 10^{-9}\;s$	2.8 · 10^−7^
(ii)			
Pb	λ	τ	*g_⋆_*
T=0K.00	$3.90\cdot 10^{-8}\;m$	$1.5\cdot 10^{-15}\;s$	1 · 10^−17^
T=4.20K	$4.3\cdot 10^{-8}\;m$	$1.8\cdot 10^{-15}\;s$	1.4 · 10^−17^
T=6.26K	$7.8\cdot 10^{-8}\;m$	$6.1\cdot 10^{-15}\;s$	8.2 · 10^−17^
T=7.10K	$2.3\cdot 10^{-7}\;m$	$5.3\cdot 10^{-14}\;s$	2.2 · 10^−15^
